# The 3-Phase Structure of Polyesters (PBT, PET) after Isothermal and Non-Isothermal Crystallization

**DOI:** 10.3390/polym14040793

**Published:** 2022-02-18

**Authors:** Dario Heidrich, Michael Gehde

**Affiliations:** Professorship of Plastics Engineering, Chemnitz University of Technology, 09126 Chemnitz, Germany; kunststoffe@mb.tu-chemnitz.de

**Keywords:** crystallization behavior, 3-phase model, polybutylene terephthalate, polyethylene terephthalate, Differential Scanning Calorimetry, Fast Scanning Calorimetry

## Abstract

According to the 3-phase model, semi-crystalline thermoplastics consist of a mobile amorphous fraction (MAF), a rigid amorphous fraction (RAF), and a crystalline fraction (CF). For the two polyesters Polybutylene Terephthalate (PBT) and Polyethylene Terephthalate (PET), the composition of these phases was investigated using the largest possible variation in the isothermal and non-isothermal boundary conditions. This was performed by combining the conventional Differential Scanning Calorimetry (DSC) with the Fast Scanning Calorimetry (FSC). From the results it can be deduced that the structural composition of both polymers is characterised by a large fraction of the rigid amorphous phase. This is mainly formed either during the primary crystallization in the low temperature range or during the subsequent secondary crystallization that follows primary crystallization in the high temperature range. Depending on the thermal history, the fraction of the mobile amorphous phase of both polymers approaches a minimum, which does not appear to be undercut.

## 1. Introduction

Despite the enormous variety of today’s polymer applications, the nature and effects of the structural characteristics of polymers are still not understood in detail. For a long period of time, the structure of semi-crystalline thermoplastics has been assumed to be a two-phase composition of an amorphous (AF) and crystalline fraction (CF). However, as investigations progressed, it was found that the assumption of the 2-phase model is not sufficient enough to describe their structural characteristics. As a result, the so-called 3-phase model was introduced, which was first verified experimentally by Menczel and Wunderlich [1] in 1981.

**Figure 1:**
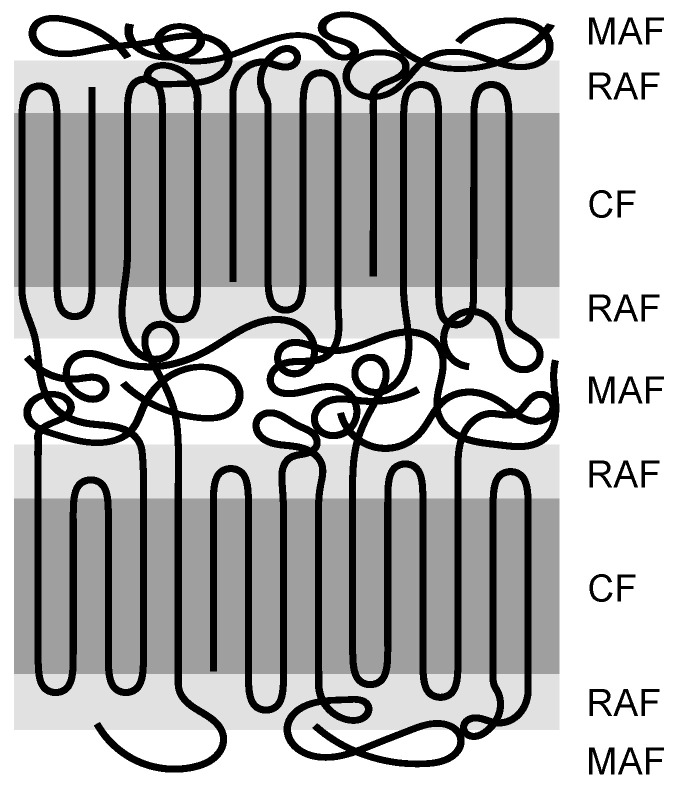
Schematic representation of the 3-phase model consisting of the mobile amorphous fraction (MAF), rigid amorphous fraction (RAF), and crystalline fraction (CF).

According to this model, the amorphous phase is further divided into a rigid and a mobile amorphous fraction (RAF, MAF). The MAF is to be equated with the conventional amorphous phase of the 2-phase model, whereas the RAF is characterised as an intermediate phase that can arise on the surface of crystallites if macromolecules do not refold properly. Thus, they are partly both inside and outside the crystallite. Because of this partial integration, the mobility of these chains is restricted compared to the ones in the mobile amorphous phase. A corresponding schematic representation of the structure of semi-crystalline thermoplastics can be seen in Figure 1.

Previous studies have shown that the primary structure of the macromolecules particularly influences the formation of the rigid amorphous phase [2]. Notably semi-rigid chain conformations lead to their formation so that, in particular, polyesters with their stiffening phenylene rings can form a distinctive rigid amorphous phase [2]. However, the three-phase structure has been proven experimentally for almost all semi-crystalline thermoplastics [3], not only with the polyesters already mentioned (e.g., PBT [4], PET [5], PC [6], PLA [7], PEN [8]) but also for example with polyamides (e.g., PA6 [9], PA66 [1]), polyolefins (PP [10], PE [11,12]) and polyacetals (POM [13]). Although there is still a lack of knowledge on the specific impact of the rigid amorphous phase, thermo-mechanical as well as physical effects could be demonstrated on the example of PET [5], PA6 [14], and PP [10].

According to Menczel [15], the typical proportion of the rigid amorphous phase is about 20–30%. However, despite the obvious importance of the three-phase characteristic, the dependence of the phase composition on the isothermal or non-isothermal boundary conditions of the crystallization was only examined within certain limits yet. For the technically important polyesters such as PBT as well as PET, the analysis of the 3-phase structure has been performed at cooling rates within the range of 1–20 K/min [4,15,16,17,18]. Isothermal crystallization experiments at individual temperatures focused on the amorphous composition of PBT were performed by Righetti and Di Lorenzo [18] as well as Cheng et al. [19]. However, only Cheng et al. [19] performed experiments over a wider range of temperatures using the DSC. Significantly more isothermal experiments were done for PET [17,20,21,22,23]. However, these are mostly based on DSC single point measurements in the high temperature range or on the cold crystallization of fully amorphous material just above the glass transition temperature. The main focus on these investigations was the temporal evolution of the phase composition, whereas a decrease in MAF resulted in an increase of the crystalline as well as of the rigid amorphous fraction.

According to the author’s knowledge, for both PET and PBT there are no extensive studies on the phase composition of the MAF, RAF, and CF under a wide variation of isothermal and non-isothermal boundary conditions. There are hardly any other studies of this kind for semi-crystalline thermoplastics in general either. Therefore, the aim in the present study was to obtain further knowledge about the structural composition of polyesters in example of PBT and PET in accordance to the 3-phase model.

## 2. Materials and Methods

### 2.1. Materials

With PBT (Ultradur^®^ B4520, BASF) and PET (Arnite^®^ A02 307, DSM Materials), two commercially available polyesters were used for the investigations. Heating with 10 K/min in the DSC, PBT shows melting at typically 223 °C and PET at 250 °C. The glass transition of PBT is in the range of 37–52 °C [24] and was assumed to be 45 °C in this study. For PET, 69 °C [24,25] was taken as transition temperature. The equilibrium melting temperatures ($T_{m}^{0}$) are subject to large differences in the literature for both polymers. For PBT the temperature of 245 °C [24] and for PET the one of 280 °C [24,25] are frequently used so that these values were assumed as well. The enthalpy of fusion of the crystalline phase is 145 J/g for PBT [26] and 140 J/g for PET [24].

### 2.2. Fast Scanning Calorimetry (FSC)

In order to investigate the isothermal as well as the non-isothermal crystallization in a preferably wide range of thermal boundary conditions, the experiments were performed with the Fast Scanning Calorimetry. More precisely, the Flash DSC 1 (Mettler Toledo) in combination with the UFS-1 sensor-chips was used. A detailed overview of the comparatively new technology of the FSC is given, for example, by Poel et al. [27].

Due to the high scanning rate of the FSC, an optimal heat transfer between the sample and sensor is particularly important. Therefore, silicone oil was used as a contact medium. In order to protect the samples from thermal decomposition, nitrogen with a flow rate of 20 mL/min was used as purge gas. To validate the results, all experiments were performed on several samples of different masses between 50 and 700 ng. Because such small sample masses are used in the FSC, their gravimetric determination was not possible. Therefore, the mass was determined by the change in heat capacity at the glass transition of a fully amorphous sample, which was achieved by quenching the melt at a cooling rate of 1000 K/s to below the glass transition. The corresponding change in the heat capacity of amorphous material was assumed to be 0.35 J/(g K) for PBT [19,24] and 0.40 J/(g K) for PET [28], respectively.

### 2.3. Differential Scanning Calorimetry (DSC)

Due to the low noise-signal ratio, the Flash DSC reaches its limits at very low cooling rates and high crystallization temperatures. In order to perform experiments under these boundary conditions as well, the conventional DSC (Q2000, TA Instruments) was used in addition. The samples with a mass between 8 and 15 mg were prepared in aluminum pans with a perforated lid. The purge gas flow of nitrogen was 50 mL/min.

### 2.4. Experimental Procedure

Referring to the standard DIN EN ISO 11357-7 [29], isothermal as well as non-isothermal experiments at constant cooling rates (CR) were performed with both polymers. By using the DSC as well as the FSC, this could be done under a very large variation in thermal boundary conditions. While the DSC was used at low cooling rates (CR < 50 K/min) as well as for the isothermal crystallization in the high temperature range ($T_{iso}$ > 200 °C), the FSC was used at correspondingly higher cooling rates or lower temperatures. For PET, cold crystallization experiments were performed with the DSC on amorphous material just above glass transition. The amorphous state was achieved by quenching the melt in liquid nitrogen.

**Figure 2:**
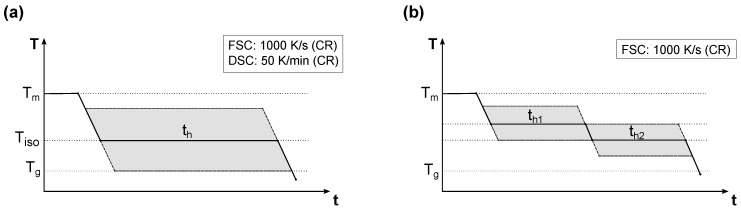
Schematic temperature curves of the crystallization experiments: (**a**) Isothermal experiments using the FSC and DSC, (**b**) subsequent isothermal crystallization at $T_{iso2}$ after an initial crystallization at $T_{iso1}$ performed with the FSC.

As shown schematically in Figure 2a, the investigations on the isothermal melt crystallization were performed starting at temperatures of 265 °C (PBT) or 290 °C (PET), respectively. In order to remove any remaining nuclei of the samples, this starting temperature was held for 2 s (FSC) or 2 min (DSC). Afterwards, the melt was cooled at 1000 K/s (FSC) or 50 K/min (DSC) to the targeted isothermal crystallization temperature $T_{iso}$ and remained there for the holding time $t_{h}$. These were chosen for each temperature by means of preliminary tests so that the primary crystallization is entirely completed. The half-crystallization times, which were investigated in one of our previous studies [30], provide a corresponding point of reference for PBT. For the experiments on the cold crystallization using the DSC, the isothermal temperature was approached at 75 K/min. After the isothermal melt crystallization as well as the cold crystallization, the samples were cooled under the glass transition at the already-mentioned cooling rates (50 K/min or 1000 K/s).

The final rapid cooling from the isothermal temperature to below the glass transition was intended to prevent any subsequent change in the structural composition due to secondary crystallization. However, in order to reconstruct this post crystallization as well, extended isothermal experiments as schematically shown in Figure 2b were performed with the FSC. The primary crystallization at the initial isothermal temperature $T_{iso1}$ was followed by a second isothermal temperature $T_{iso2}$ at a lower level. The first holding time $t_{h1}$ was again chosen so that the primary crystallization at $T_{iso1}$ is complete. For the second temperature $T_{iso2}$, the holding time $t_{h2}$ was varied in order to detect the temporal evolution of the secondary crystallization (PBT: up to 400 s; PET: up to 3600 s).

**Figure 3:**
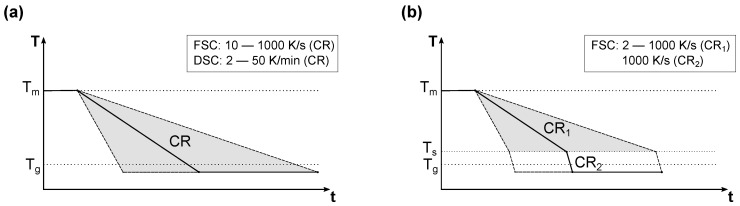
Schematic temperature curves of the non-isothermal crystallization experiments: (**a**) conventional non-isothermal experiments at constant cooling rates (CR) using the FSC and DSC in order to determine $X_{CF}$, (**b**) non-isothermal experiments for the determination of $X_{MAF}$ using the FSC. Therefore, the cooling rate is changed at $T_{s}$ in order to prevent kinetic influences on the subsequent heating.

In the non-isothermal experiments, the crystallization behavior from the melt to below the glass transition was investigated using constant cooling rates in the range from 2 K/min to 1000 K/s (Figure 3a). For this, both the DSC and the FSC were used again.

In the illustrated experimental procedures, the crystallinity $X_{CF}$ was determined differently for the two materials. In the case of PBT, this was done directly from the exothermic crystallization peak, regardless of whether the DSC or the FSC was used. Due to the low signal-to-noise ratio caused by the low crystallization rate of PET during isothermal as well as non-isothermal experiments, this was not possible for the measurements with the FSC. The crystallinity of PET was therefore determined using the enthalpy of fusion during the subsequent heating. This subsequent heating was performed anyway after all the mentioned crystallization experiments in order to determine the fraction of the mobile amorphous phase ($X_{MAF}$) from the change in the heat capacity at the glass transition through the following equation:

(1) $$X_{MAF}=\frac{\Delta c_{p}}{\Delta c_{p-am}}$$

where $\Delta c_{p}$ is the measured change in specific heat capacity of the semi-crystalline sample at the glass transition during the heating and $\Delta c_{p-am}$ is the change in the specific heat capacity of the fully amorphous material. As already mentioned, the latter one is 0.35 J/(g K) for PBT [19,24] and 0.40 J/(g K) for PET [28], respectively. In experiments with the DSC, the reheating was performed by temperature modulation (TMDSC) so that the determination of $\Delta c_{p}$ was done on the reversing signal. This made it possible to negate disturbing influences such as cold crystallization. Therefore, the heating rate of 2 K/min was superimposed with a sinusoidal oscillation with an amplitude of 0.5 K (PBT) or 1.0 K (PET) and a period of time of 60 s. In the isothermal crystallization experiments using the FSC, the subsequent heating was performed at 1000 K/s.

In the case of reheating the non-isothermal experiments afterwards using the FSC with the mentioned scanning rate, there is a large difference between the previous cooling and the following heating rate. As Menczel and Wunderlich [1] have shown using the example of PET, this can have an effect on the glass transition as a kinetic phase transition. Thus, this can lead to inaccuracies in the analysis of $X_{MAF}$. Due to this, the non-isothermal experiments for the determination of the mobile amorphous fraction were adapted based on the investigations by Parodi et al. [14] (Figure 3b). After the cooling at a constant first cooling rate (CR1), immediately before the glass transition a switch was made to the cooling rate of 1000 K/s (CR2). Therefore, the scanning rates during cooling and subsequent heating were identical at the glass transition so that additional kinetic effects were prevented. After preliminary tests, the switch temperature $T_{s}$ was set to 90 °C for PBT and 110 °C for PET. Once both the crystallinity $X_{CF}$ and the mobile amorphous fraction $X_{MAF}$ are known, the fraction of the rigid amorphous phase $X_{RAF}$ can be calculated using the following equation:

(2) $$X_{RAF}=1-X_{MAF}-X_{CF}$$

## 3. Results and Discussion

### 3.1. Phase Composition after Isothermal Crystallization

**Figure 4:**
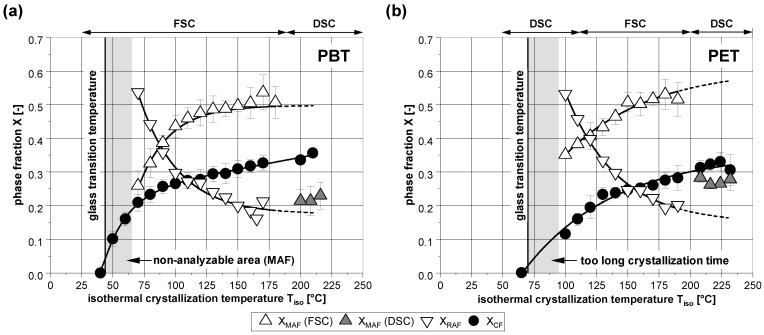
Determined phase composition of (**a**) PBT and (**b**) PET in dependence on the isothermal crystallization temperature. The lines are for visual purposes only.

Considering the 3-phase model, in Figure 4 for PBT and PET the phase composition after isothermal crystallization is shown. As expected, the crystallinity ($X_{CF}$) of both polymers increases with higher crystallization temperatures. If the phase composition is analysed in regard to the 2-phase model, this would result in a decreasing cumulative amorphous phase for both polymers. Separating the amorphous phase into its two components according to the 3-phase model, a different course can be seen. Starting from the glass transition temperature, the mobile amorphous fraction ($X_{MAF}$) increases significantly with higher crystallization temperatures. In contrast, the rigid amorphous fraction ($X_{RAF}$) decreases sharply.

As shown by the greyed out areas, there are some limitations to the analyzability for both polymers within the low temperature range. In the case of PBT, during the heating after the isothermal crystallization at low temperatures, a distinctive cold crystallization occurs just above the glass transition. Thus, the change in the heat capacity and as a result, the fraction of the amorphous phase cannot be determined. In the case of PET, the crystallization times in the marked low temperature range are very long and were therefore not subject of the present study.

Furthermore it is noticeable that the fraction of the mobile amorphous phase determined by (TM)DSC after the isothermal crystallization at high temperatures does not match to the general curve shape (grey triangles) for both polymers. The level of the values seems generally too low. The reason for this is presumably the secondary crystallization which occurs during the subsequent slow cooling with the DSC, as described in Section 3.2.

**Figure 5:**
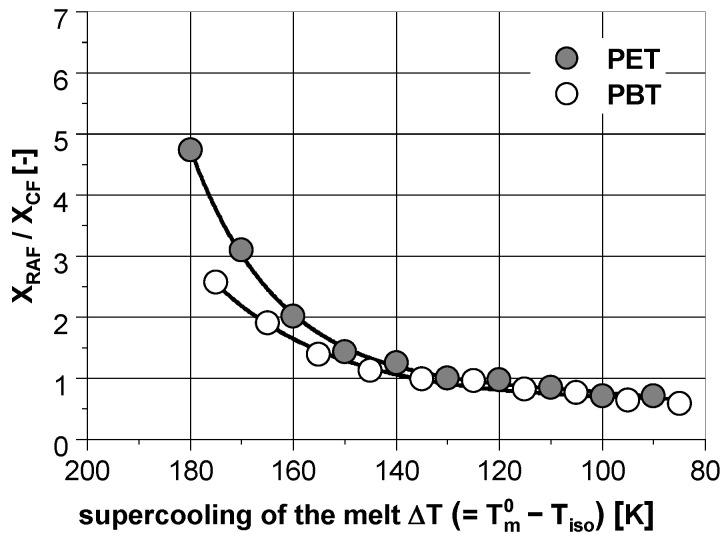
Specific rigid amorphous fraction ($X_{RAF}$/$X_{CF}$) in dependence on the isothermal supercooling of the melt for PET and PBT, derived from the data in Figure 4. The lines are for visual purposes only.

All in all, due to the greater mobility of the macromolecules and based on the better refolding of the molecular chains, the formation of crystallites at high temperatures is associated with less structural defects. As shown in Figure 5, this is particulary evident by the representation of the specific RAF ($X_{RAF}$/$X_{CF}$). With increasing undercooling of the melt, this value increases significantly. Presumably due to a more rigid primary structure of PET [31], their increase is greater than in PBT. However, with both polymers it becomes clear that at low temperatures, the mobile amorphous phase is mainly converted into the rigid amorphous phase during the crystallization.

**Figure 6:**
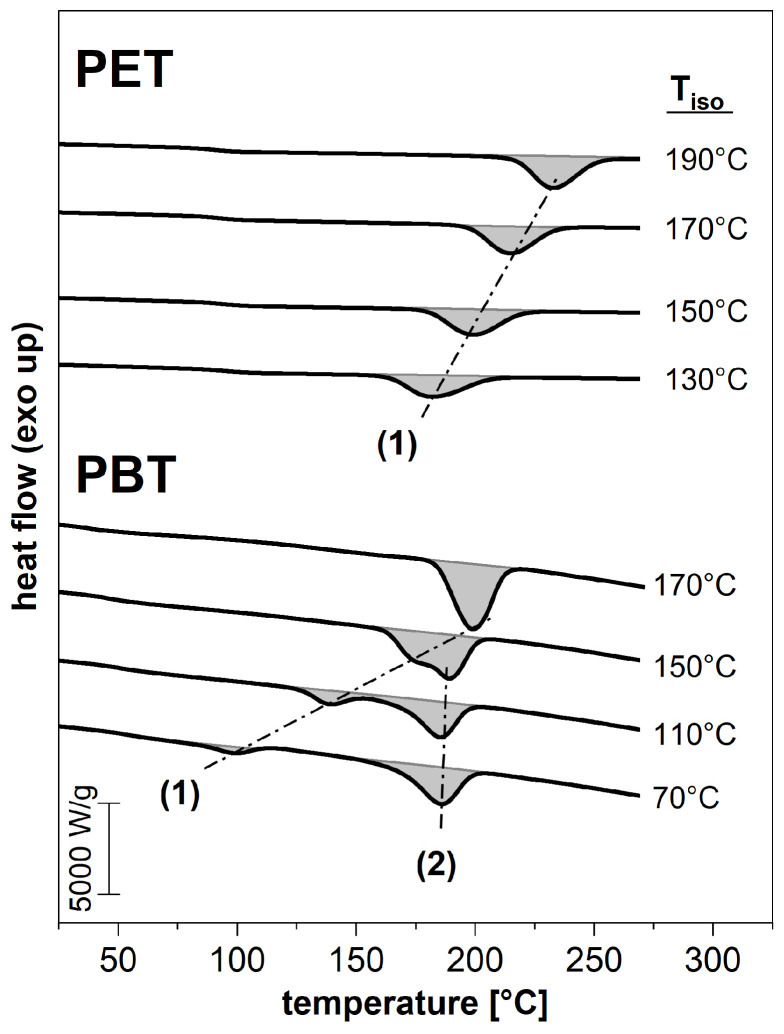
Exemplary first heating runs (at 1000 K/s) after the isothermal crystallization at $T_{iso}$ obtained with the FSC.

Subsequently, the melting behavior resulting from the isothermal crystallization was investigated. The first heating at 1000 K/s after isothermal crystallization with the FSC is exemplary shown for some isothermal crystallization temperatures in Figure 6. In the case of PET, after the glass transition a melting peak (1) appears that depends on the previous crystallization temperature $T_{iso}$. Thus, with increasing prior crystallization temperature, it shifts to higher temperatures. Due to the high heating rate of the FSC and the associated thermal lag, the shift between these two temperatures ($T_{iso}$ and $T_{m}$) for PET is around 45 K. The same applies to PBT, with the shift being slightly less at about 30 K.

In the case of PBT, the occurrence of a further melting peak (2) at around 190 °C is noteworthy, which initially is independent on the thermal history. It is known from other studies [32,33,34] that this melting is based on the reorganization during the heating of the measurement itself. Since the endothermic melting of existing crystallites is superimposed with the simultaneous exothermic recrystallization with equal intensity, the resulting effect cannot be seen in the heat flow signal [35]. Furushima et al. [34] have shown that this reorganization of continuous melting and crystallization of PBT can only be suppressed with a heating rate of 100,000 K/s. With a higher crystallization temperature, melting (1) gradually passes into melting (2). From an isothermal crystallization temperature of 170 °C, just one melting peak exists. Once again, this is directly related to the prior formation temperature and is thus associated with melting (1).

### 3.2. Subsequent Secondary Crystallization

As indicated, the mobile amorphous fraction remaining after the primary crystallization at high temperatures is large for both polymers. This ensures great potential for secondary crystallization during the subsequent cooling, in particular in case of the comparatively slow maximum cooling rate of the DSC. As a result, the determined fraction of the mobile amorphous phase after the crystallization at high temperatures measured with the DSC is too low, as already shown in Figure 4. However, the subsequent change in the structural composition can obviously be prevented using the FSC with its high cooling rate.

**Figure 7:**
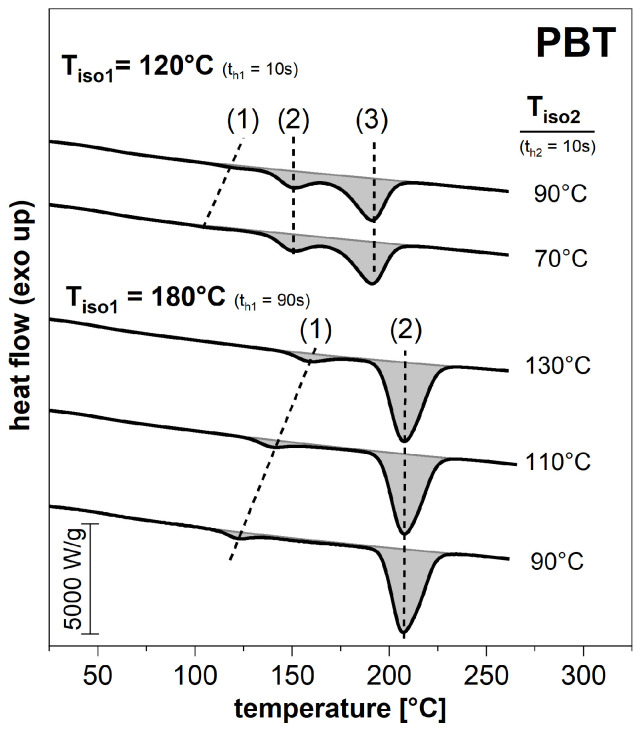
Exemplary first heating runs (at 1000 K/s) after the primary and subsequent secondary crystallization at $T_{iso1}$ and $T_{iso2}$, respectively, obtained with the FSC. The holding time at $T_{iso1}$ was 10 s (120 °C), respectively, 90 s (180 °C), and 10 s at $T_{iso2}$.

For further investigations with the FSC, the primary isothermal crystallization at $T_{iso1}$ was followed by an isothermal temperature $T_{iso2}$. Due to the relatively small effect and the correspondingly low signal-to-noise ratio, the occurring secondary crystallization could not be traced directly. However, it could be detected indirectly during the subsequent heating through the additional melting effects. Figure 7 compares the heating of PBT after an additional secondary crystallization at different temperatures, if the previous primary crystallization was performed at 120 °C or 180 °C, respectively.

During the subsequent heating, a melting peak (1) appears, which seems to be related to the crystallization at the second isothermal temperature $T_{iso2}$. This occurs in addition to the melting (2), which is related to the primary crystallization at the temperature $T_{iso1}$. Since $T_{iso1}$ in case of 120 °C is lower than 170 °C, the melting peak (3) is attributed to the reorganization during heating, as already shown before.

**Figure 8:**
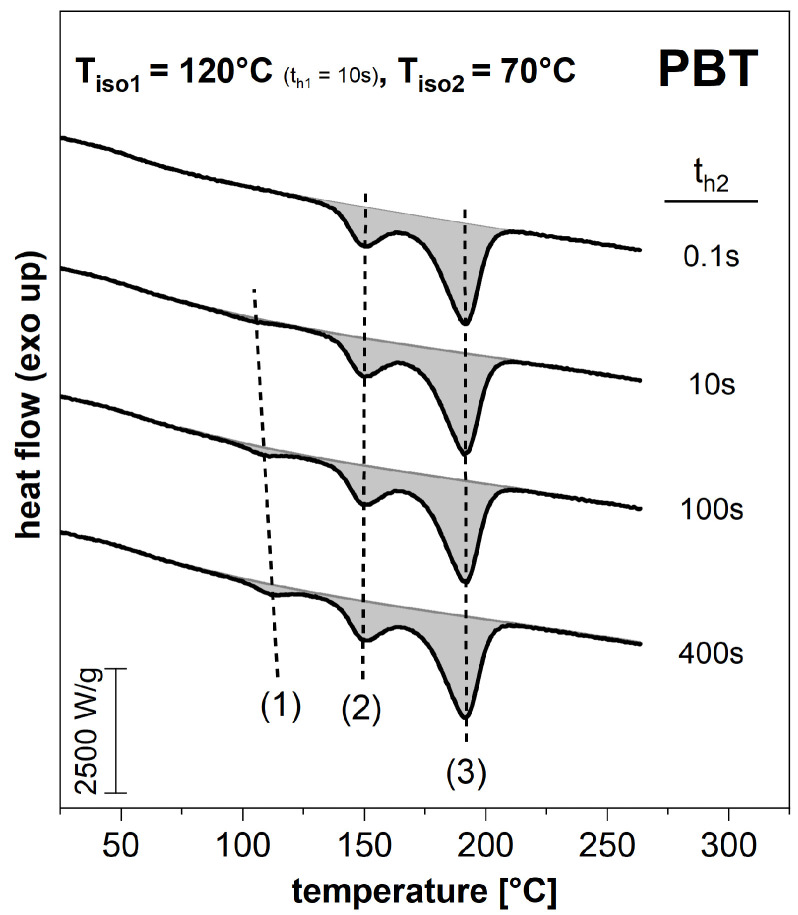
Exemplary first heating runs (at 1000 K/s) after the primary and subsequent secondary crystallization at 120 °C and 70 °C, respectively, obtained with the FSC. The holding time $t_{h}$ at 120 °C was 10 s, whereas it varied at 70 °C in the range from 0.1 to 400 s.

The results shown so far are based on a holding time $t_{h2}$ of the subsequent crystallization of 10 s each. In further experiments, this holding time was gradually varied in the range from 0.1 to 400 s. Using the example of a first crystallization at $T_{iso1}$ = 120 °C and a subsequent crystallization at $T_{iso2}$ = 70 °C, the resulting heating curves are shown in Figure 8. It can be seen that the intensity of the melting peak (1), which is again related to secondary crystallization, increases the longer the corresponding holding time. At the same time, the melting shifts to higher temperatures. Thus, it can be seen that the rate of secondary crystallization is quite low in respect to the primary one. The melting peaks (2) and (3) in Figure 8 are once again related to the primary crystallization at $T_{iso1}$ and the reorganization during heating, respectively.

**Figure 9:**
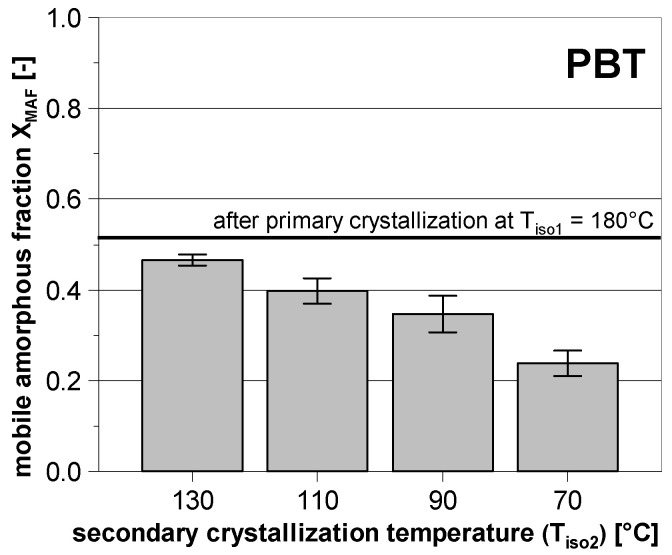
Evolution of the mobile amorphous fraction of PBT after secondary crystallization at different temperatures subsequent to the primary crystallization at 180 °C. The corresponding holding times were 90 s (180 °C) and 400 s (130–70 °C), respectively.

Due to the superimposed melting and recrystallization during the heating of PBT, it is not useful to determine the additional crystallinity of the secondary crystallization using the corresponding melting enthalpy. However, in addition to the change in crystallinity, the secondary crystallization influences the composition of the amorphous phase as well. Figure 9 shows exemplary how the fraction of the mobile amorphous phase ($X_{MAF}$) of PBT decreases, if the primary crystallization at 180 °C is followed by a secondary crystallization at 130–70 °C for a period of time of 400 s. It can be seen that $X_{MAF}$ clearly decreases with decreasing secondary crystallization temperature. This shows that a distinctive secondary phase transition can also occur briefly above the glass transition. Furthermore, most of the mobile amorphous fraction will be converted into the rigid amorphous phase.

**Figure 10:**
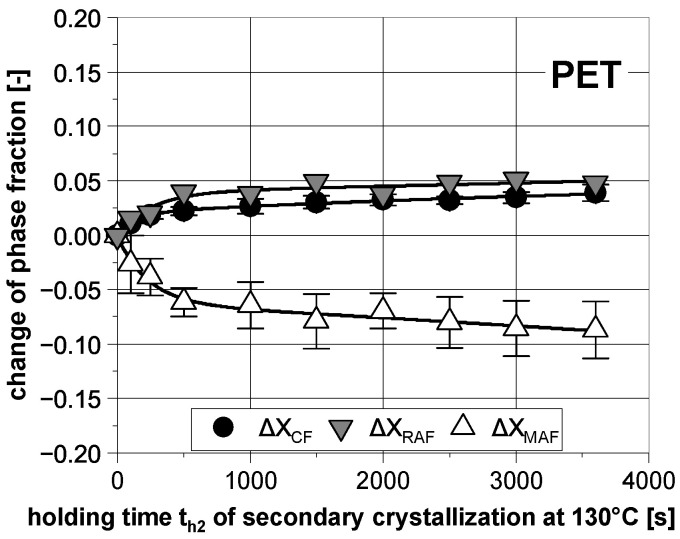
Temporal evolution of the change of phase fractions of PET during secondary crystallization at 130 °C subsequent to the primary crystallization at 180 °C ($t_{h1}=2000$ s). The lines are for visual purposes only.

The experimental procedure to investigate the isothermal secondary crystallization was applied to PET on a random basis for the combination of 180 °C ($T_{iso1}$) and 130 °C ($T_{iso2}$) as well. The holding time at $T_{iso1}$ was constant at 2000 s, whereas it was varied at $T_{iso2}$ (up to 3600 s). As with PBT, at the subsequent heating a further melting peak occurred, which can be attributed to the prior secondary crystallization. Based on the determined additional melting enthalpy and the change of glass transition, in Figure 10 the temporal evolution of the phase fractions caused by the subsequent crystallization at 130 °C in dependence on the corresponding holding time $t_{h2}$ is presented.

It becomes clear that the initial mobile amorphous phase is converted into both the rigid amorphous phase and the crystalline phase over a long period of time. The temporal evolution of the additional crystallinity is in a $\sqrt{t}$-relation, which proves the interpretation as secondary crystallization [36].

### 3.3. Phase Composition after Non-Isothermal Crystallization

**Figure 11:**
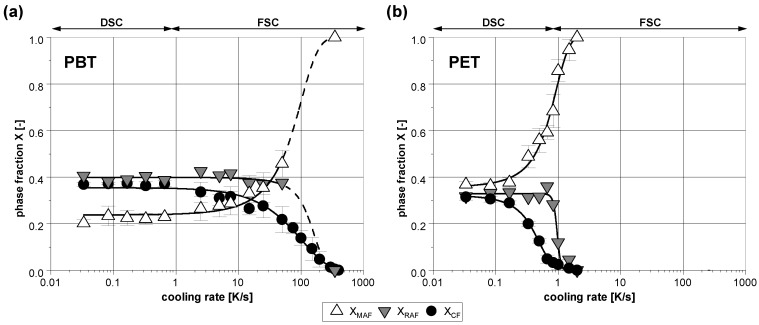
Determined phase composition of (**a**) PBT and (**b**) PET in dependence on the cooling rate performed with DSC and FSC. The lines are for visual purposes only.

In the case of PBT, the determination of the phase composition resulting from the non-isothermal experiments is a challenge since the first heating after high cooling rates results in a distinctive cold crystallization immediately after the glass transition. Therefore, the analysis of the mobile amorphous phase was only possible to a limited extent up to a cooling rate of around 50 K/s (Figure 11). As expected, the crystallinity $X_{CF}$ of both polymers decreases with increasing cooling rate. From a cooling rate of 350 K/s (PBT) or 2 K/s (PET), the respective crystallization is completely suppressed. The fraction of the mobile amorphous phase has, in each case, a minimum at around 0.2 (PBT) or 0.4 (PET), respectively. Due to the suppressed crystallization, the fraction of the mobile amorphous phase ($X_{MAF}$) increases significantly with increasing cooling rate. The fraction of the rigid amorphous phase ($X_{RAF}$) is comparatively less influenced by the applied cooling rate for both polymers. It is almost constant and drops sharply when crystallization is almost completely suppressed.

Considering the 3-phase composition resulting from the non-isothermal crystallization, there is an alleged contradiction to the results of the isothermal experiments for both of the polymers investigated. As expected, the fraction of the crystalline phase decreases with increasing cooling rate. In contrast to this, the fraction of the mobile amorphous phase increases. Since a higher cooling rate results in a lower crystallization temperature, it has to be expected that the fraction of the mobile amorphous phase decreases as well (in regard to the isothermal crystallization results). Likewise, the fraction of the rigid amorphous phase has to increase significantly with increasing cooling rates. Instead, it remains almost independent on the cooling rate for both polymers.

To solve this contradiction, the knowledge of the isothermal experiments and the associated secondary crystallization during the subsequent cooling must be taken into account. During the non-isothermal experiments at low cooling rates, the primary and secondary crystallization are superimposed. Low cooling rates result in a distinctive primary crystallization at high temperatures, which then offers great potential for secondary crystallization at lower temperatures. Therefore, the associated continuous crystallization during slow cooling not only results in high crystallinity, but also in a high fraction of the rigid amorphous phase. On the other hand, at high cooling rates the formation of the crystalline phase is much more inhibited than with isothermal crystallization, in which the respective temperature was held until the primary crystallization was fully completed.

### 3.4. Comparison to the Literature

**Figure 12:**
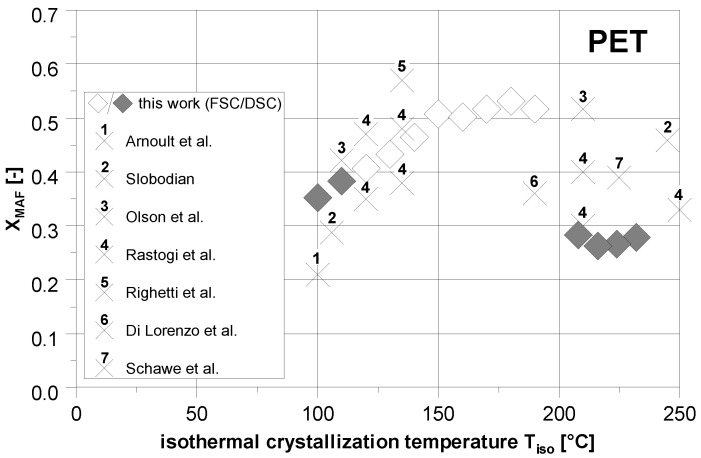
Comparison of the determined $X_{MAF}$ to the literature regarding the isothermal crystallization of PET. 1—[23]; 2—[21]; 3—[37]; 4—[5]; 5—[16]; 6—[22]; 7—[20].

As already mentioned, a comparison of our results with data from the literature is difficult since the thermal boundary conditions of crystallization for both polymers were varied only to a limited extent. However, if the individual measurements of various authors on PET are combined as shown in Figure 12, the fraction of the mobile amorphous phase increases with the crystallization temperature as well. Our own results can meaningfully supplement the literature values and thus confirm the basic course of the $X_{MAF}$ in case of the isothermal crystallization of PET. At the same time, it becomes clear that some results of other authors show a decrease in the mobile amorphous fraction within the high temperature range as well. Likewise, their applied cooling rate seems to be too low to prevent a subsequent change in the materials structure. However, it must be noted that different commercial products were used in the various studies and therefore the crystallization behavior may also differ slightly.

For PBT, a similar trend of $X_{MAF}$ was determined in our study. However, this is contrary to the results of Cheng et al. [19]. Their fraction of the mobile amorphous phase is almost independent of the crystallization temperature with a value of approximately 0.2. As stated, our DSC measurements at high temperatures result in corresponding values as well. As it could be shown within the scope of this work, this is presumably based on the influence of the secondary crystallization during the subsequent (slow) cooling with the DSC and the associated conversion of the MAF mainly into RAF.

Even with the results of the non-isothermal experiments, a comparison with the literature in relation to the 3-phase model is possible to a limited extent as well, since comparative values are only available for the very low cooling rates of the DSC. For PET, Menczel [15] determined a composition of the amorphous phases ($X_{MAF}$, $X_{RAF}$) at 1 K/min of 0.45/0.18 and Righetti et al. [17] at 2 K/min a one of 0.41/0.18. In the case of PBT, Menczel [15] investigated a composition of 0.22/0.41 at 2 K/min as well as a composition of 0.28/0.41 at 20 K/min. Even if this only allows a selective comparison in case of very low cooling rates, our own results in this range match the literature values.

## 4. Conclusions

For both polyesters, the results show that in the low temperature range the phase transition is accompanied by a presumably high density of defects. Therefore, the rigid amorphous fraction remaining after the primary crystallization is very high and that of the mobile amorphous one is quite low. If the melt is just slightly supercooled, this effect is reversed due to a presumably much better refolding of the macromolecules. This results in an increasing mobile amorphous fraction with the crystallization temperature. The comparison with studies of individual measurements from the literature has confirmed this trend.

The large fraction of the mobile amorphous phase remaining after the crystallization at high temperatures offers great potential for a secondary crystallization at subsequent lower temperatures. Since at high supercooling the specific rigid amorphous fraction ($X_{RAF}$/$X_{CF}$) is well above one for both polymers investigated, the remaining mobile amorphous phase is just slightly converted into the crystalline phase at these low temperatures. Rather, the rigid amorphous phase is primarily formed.

For non-isothermal cooling, this means that a large proportion of RAF is almost always formed, regardless of the exact thermal history—either through primary crystallization at low temperatures (in case of a high cooling rates) or through a combination of primary and secondary crystallization (in case of low cooling rates). Thus, only at very high cooling rates at which crystallization is already strongly suppressed can the rigid amorphous fraction be strongly influenced.

## Data Availability

The raw/processed data required to reproduce these findings cannot be shared at this time as the data also forms part of an ongoing study.
